# Dynamics in the prevalence and clinical manifestations of acute mountain sickness of different ascent protocols during high altitudes exposure

**DOI:** 10.3389/fpubh.2024.1472935

**Published:** 2024-11-21

**Authors:** Caitong Zhao, Xinyu Zhao, Yan Ma, Yupeng Liu, Renzheng Chen, Lide Sha

**Affiliations:** ^1^Department of Quality Control, General Hospital of Northern Theater Command, Shenyang, China; ^2^Department of Critical Care Medicine, The 967th Hospital of Joint Logistics Support Force of Chinese PLA, Dalian, China; ^3^The Second Medical Center and National Clinical Research Center for Geriatric Diseases, Chinese PLA General Hospital, Beijing, China; ^4^Department of Emergency, The 967th Hospital of Joint Logistics Support Force of Chinese PLA, Dalian, China; ^5^Department of Gastroenterology, The 967th Hospital of Joint Logistics Support Force of Chinese PLA, Dalian, China

**Keywords:** high altitude, acute mountain sickness, ascent protocol, risk factor, clinical manifestations

## Abstract

**Background:**

Leisure, work, and sports activities that involve ascending to high altitudes (HA) are growing in popularity, yet they also pose the risk of developing acute mountain sickness (AMS). Despite the dynamic nature of AMS, its prevalence, clinical manifestations, and associated risks have still not to be comprehensively characterized.

**Methods:**

A total of 770 healthy males, ranging in age from 18 to 45 years, were included in this study. The subjects were divided into two cohorts: a fast ascent cohort (*n* = 424) who ascended to 3,650 m by airplane, and a slow ascent cohort (*n* = 346) who ascended to the same altitude by bus. Subsequently, they all further ascended to 4,400 m. AMS was diagnosed using the Lake Louise Scoring system (LLS), with either the old or new version were employed.

**Results:**

As diagnosed by the old LLS and new LLS, the incidence of AMS was 37.9 and 32.4% at 3650 m, respectively, which decreased to 35.7 and 32.4% after further ascending to 4,400 m in the fast ascent cohort; the incidence of AMS was 26.5 and 23.2% at 3650 m, which increased to 44.5 and 42.3% after further ascending to 4,400 m in the slow ascent cohort. Furthermore, there were noticeable disparities in the occurrence and progression of AMS-related symptoms among cohorts adhering to different ascent protocols. Specifically, fast ascent protocol posed a risk during the initial phase of the ascent, but transformed into a protective effect upon further ascent to a higher altitude.

**Conclusion:**

Ascent protocol emerged as the pivotal influence on the prevalence of AMS and associated manifestations, demonstrating a transition from a risk factor during initial ascent to a protective factor following further ascent to higher altitudes. These findings suggest an innovative strategy for high-altitude expeditions and work endeavors, emphasizing the importance of a strategic plan for ascending to higher altitudes.

## Introduction

The number of sea-level inhabitants engaging in leisure, work, or sports activities at high altitudes (HA) is rapidly increasing. However, during the initial days of arrival at HA exceeding 2,500 m, a substantial proportion of individuals who are not adequately acclimatized may experience acute mountain sickness (AMS), contingent upon the attained altitude, the pace of ascent, individual vulnerability, and the extent of acclimatization (1, 2). If not treated appropriately, AMS may deteriorate into severe health risks, including high-altitude pulmonary oedema (HAPE) and high-altitude cerebral oedema (HACE) (3), both were life-threatening, and could hinder the development of social economy (4). Thus, it is of paramount importance to illuminate the prevalence and risks of AMS in order to prevent it, as it significantly impacts the health and quality of life of millions of travelers and workers in HA.

AMS symptoms are generally non-specific yet debilitating, featuring headaches, gastrointestinal (GI) upset, fatigue, dizziness, insomnia, and a myriad of other symptoms (5). However, insomnia, due to its poor correlation with the other hallmark symptoms of AMS, has been excluded as the defining criterion for its diagnosis under the revised Lake Louise Scoring (LLS) system, which was updated in 2018 (6). Nevertheless, there is still a relative lack of information on the prevalence and clinical manifestations of AMS as defined by LLS, and the difference in diagnostic effectiveness between the old and new versions also remains controversial (7).

Identifying the associated risk factors of AMS is crucial, as a prior study has revealed that the primary determinants of its development were the altitude reached and the rate of ascent. Specifically, the likelihood of experiencing AMS escalated with increasing altitudes, and a faster pace of ascent significantly increased the risk (8–10). Additionally, some factors, such as age, sex, body mass index (BMI), smoking and genetics, have been demonstrated to be associated the development of AMS (11–14). Moreover, other previous studies have also demonstrated a decline in the progression of AMS when subjected to pre-acclimatization procedures (such as physical exercise before climbing) (15, 16) or extended acclimatization schedules (such as staying at medium altitude for days) (17, 18). When individuals ascend to HA, they do not always have a definitive endpoint. Commonly, after a temporary stay at an intermediate altitude, they may choose to continue upwards for a variety of reasons. This means that the prevalence and clinical manifestations of AMS may undergo significant shifts over time. Furthermore, final AMS-related disease status may also vary depending on different ascending strategies employed before reaching higher altitudes. Early exposure to a certain altitude at a rapid rate, despite the heavy risk of AMS, may affect the level of acclimation and offer benefits for further ascent. Nevertheless, these dynamics and the ideal ascent protocols have yet to be fully explored and documented.

For these reasons, the objectives of the present study were to reveal the dynamics of the prevalence and clinical manifestations of AMS diagnosed by both the old and the new LLS in a large population ascending to heights above 3,000 m with a fast or a slow ascent protocol, with subsequent ascending to elevations exceeding 4,000 m. In addition, to compare the status of AMS-related diseases at higher altitudes across various ascent protocols in this situation, thereby offering clinical insights to inform the development of suitable plans for ascending to high altitudes in the future.

## Methods

### Study population and ethical considerations

A total of 770 healthy males who had spent most of their lives in the lowland (<500 m, above sea level [SL]) were included in this retrospective study. They performed a comprehensive medical examination before the expedition in June 2023. Subjects with any clinical conditions that may exhibit HA-related symptoms were excluded, including known pulmonary diseases, cardiovascular diseases, haematologic diseases, liver and kidney dysfunction, malignant tumors, and so on. Subjects with psychiatric disorders that prevented the completion of the data collection and subjects with HACE or HAPE needing emergency treatment were also excluded. This study was approved by the Human Ethics Committee of the 967th Hospital of Joint Logistics Support Force of Chinese PLA (No. PLA967-GC2023-021) and was conducted in agreement with the Declaration of Helsinki. All subjects volunteered to participate in this study and gave written informed consent.

### Ascending procedures

**Figure 1:**
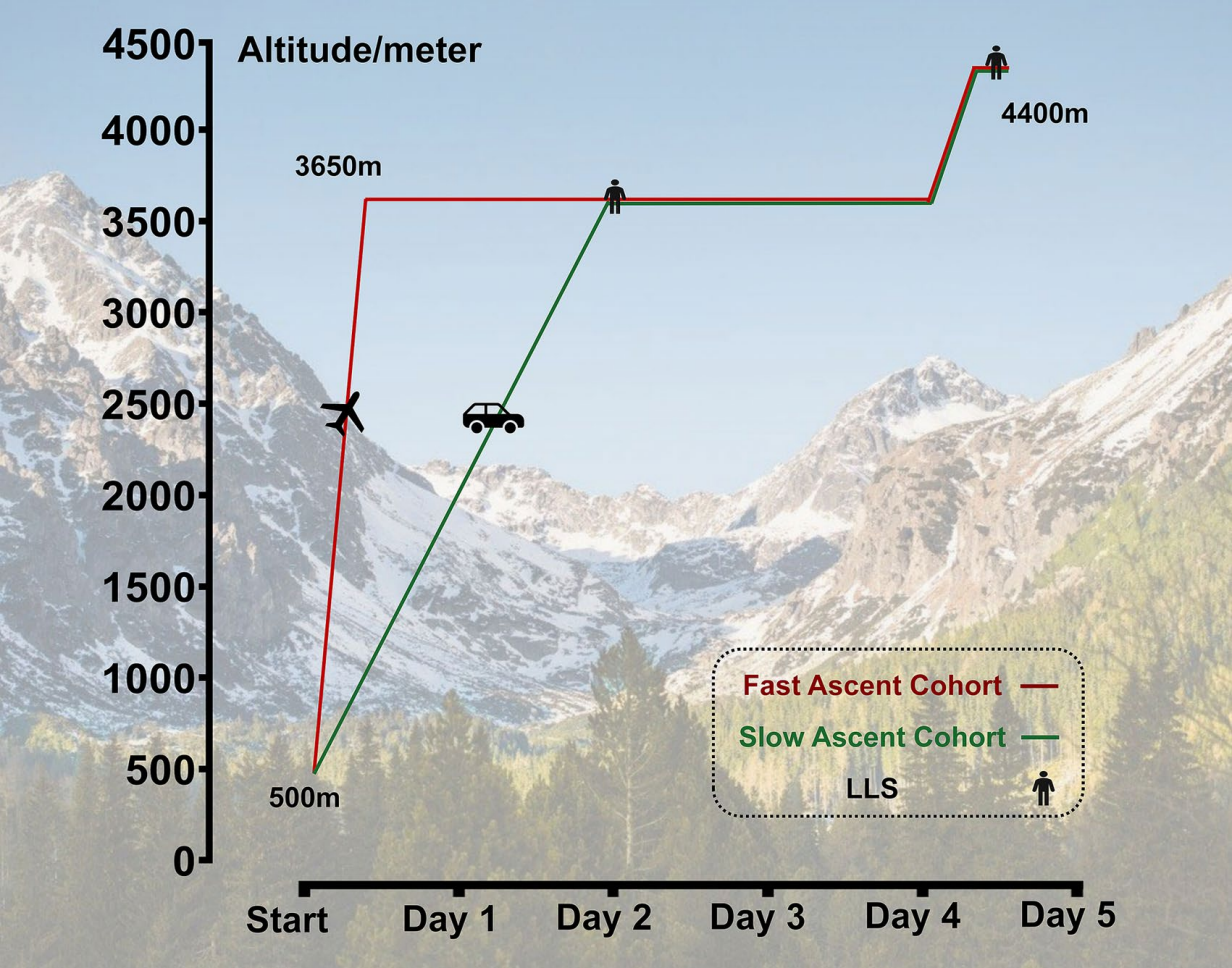
Study flow chart.

All subjects successively ascended to HA from 500 m (Chengdu, Sichuan, China) to 3,650 m (Lhasa, Tibet, China) by airplane in approximately 3 h or by bus within 2 days in a staged ascent mode in July 2023. After staying for 2 days at 3650 m, they further ascended to 4,400 m (Yangbajin, Tibet, China) by bus. Among them, 53 subjects who refused to ascend further to 4,400 m and/or with incomplete questionnaires were excluded. Ultimately, the data from 717 subjects who successfully reached 4,400 m was collected. For subjects who quickly ascended to the plateau by air, we named them as the “fast ascent cohort,” correspondingly, those who ascended to the plateau by bus at a slower speed were referred to as the “slow ascent cohort.” This classification was based on different modes of transportation and arrival speeds (Figure 1).

### Data collection

Data collection was performed on June 2023 at SL and 12–24 h after arriving at 3650 m or 4,400 m. All subjects were instructed to refrain from engaging in unusual physical activities and to adhere to a standard schedule. They were required to abstain from smoking and alcohol consumption, and medication was only permitted for special treatment needs. Structured case report form questionnaires were used to record demographic information, including age, BMI, smoking, nationality, educational level and history of HA exposure within 1 year. Clinical symptoms were self-reported, including headache, dizziness, GI upset, insomnia, fatigue, paraesthesia, constipation, dyspnoea, cough, chest distress, palpitation, tinnitus, dazzling, lethargy, and reduced activity.

### Diagnosis of AMS

The standardized LLS has been employed to reliably establish the incidence of AMS (5). AMS-related symptoms including: headache, dizziness, GI upset (e.g., nausea), fatigue, and insomnia. The severity of the AMS symptoms was scored from 0 (no discomfort) to 3 (severe discomfort) according to the subjects’ self-report. The old and the new LLS-AMS were both used (6). Accordingly, subjects with LLS ≥ 3 in the presence of headache were diagnosed with AMS.

### Statistical analysis

All the statistical analyses were performed using SPSS 27.0 for Windows (IBM Corp., Armonk, NY, USA). Demographic characteristics, clinical symptoms and the incidence of AMS were presented as the mean ± standard deviation for continuous variables and compared using Student’s t tests or the Mann–Whitney U test according to their normality following the Kolmogorov–Smirnov test. Categorical variables were expressed as counts and percentages and analysed using the *χ*^2^ test or Fisher’s exact test, as appropriate. For the risk factor analysis, univariate and multivariate logistic regression analysis was performed. Age, BMI, smoking, nationality, education and history of HA exposure were adjusted in the multivariate analysis. Moreover, to reduce the effect of potential confounding factors in the present study, propensity score-matching was performed for rigorous adjustment for significant differences in the baseline characteristics. Statistical power was calculated by PASS software (version 11, NCSS, LLC, Kaysville, UT, USA), and more than 80% was achieved between subgroups using a two-sided alpha of 0.05. All the statistical tests were two-sided, and a *p*-value of <0.05 was considered statistically significant.

## Results

### Subject characteristics

Upon reaching an altitude of 3,650 m, 251 subjects (32.6%) were diagnosed with AMS using the old LLS, whereas 222 subjects (28.8%) were diagnosed with AMS utilizing the new LLS. Similarly, at an altitude of 4,400 m, 277 subjects (36.0%) were diagnosed with AMS according to the old LLS, compared to 257 subjects (33.4%) who were diagnosed with AMS using the new LLS. Subjects were grouped according to whether they were diagnosed AMS or not. The baseline demographic characteristics of the subjects are shown in Tables 1, 2. No significant differences were found in Age, BMI, history of smoking, nationality, education level, and history of HA exposure between the two groups. While the AMS group exhibited a higher proportion of subjects demonstrating fast ascent at 3650 m (61.8% vs. 51.8%, *p* = 0.009, 61.7% vs. 52.4%, *p* = 0.018), it was noteworthy that, at 4400 m, the percentage of subjects exhibiting a fast ascent was comparatively lower in those suffering from AMS than those who did not experience AMS (50.5% vs. 59.8%, *p* = 0.015, 49.0% vs. 60.2%, *p* = 0.004).

**Table 1:** Baseline demographic characteristics in AMS (+) and AMS (−) subjects after arriving at 3650 m.

	Old LLS		*p* value	New LLS		*p* value
	AMS (+) (*n* = 251)	AMS (−) (*n* = 519)		AMS (+) (*n* = 222)	AMS (−) (*n* = 548)	
Age, years	21.26 ± 3.03	21.32 ± 3.02	0.783	21.05 ± 2.68	21.41 ± 3.15	0.140
BMI, kg.m^−2^	21.29 ± 1.87	21.23 ± 1.83	0.655	21.21 ± 1.76	21.27 ± 1.87	0.699
Nationality, *n* (%)			0.239			0.303
Han people	226 (90.0%)	469 (90.4%)		199 (89.6%)	496 (90.5%)	
Tibetan	2 (0.8%)	12 (2.3%)		2 (0.9%)	12 (2.2%)	
Others	23 (9.2%)	38 (7.3%)		21 (9.5%)	40 (7.3%)	
Smoking, *n* (%)			0.964			0.550
Non	66 (26.3%)	141 (27.2%)		54 (24.3%)	153 (27.9%)	
Previous	59 (23.5%)	122 (23.5%)		56 (25.2%)	125 (22.8%)	
Current	126 (50.2%)	256 (49.3%)		112 (50.5%)	270 (49.3%)	
History of HA exposure within 1 year, *n* (%)			0.690			0.598
Yes	41 (16.3%)	79 (15.2%)		37 (16.7%)	83 (15.1%)	
No	210 (83.7%)	440 (84.8%)		185 (83.3%)	465 (84.9%)	
Ascent rate, *n* (%)			0.009			0.018
Fast	155 (61.8%)	269 (51.8%)		137 (61.7%)	287 (52.4%)	
Slow	96 (38.2%)	250 (48.2%)		85 (38.3%)	261 (47.6%)	
Education, *n* (%)			0.878			0.669
University	45 (17.9%)	101 (19.5%)		38 (17.1%)	108 (19.7%)	
High school	145 (57.8%)	295 (56.8%)		128 (57.7%)	312 (56.9%)	
Under high school	61 (24.3%)	123 (23.7%)		56 (25.2%)	128 (23.4%)	

Values are mean ± SD or *n* (%); AMS, acute mountain sickness; BMI, Body Mass Index; HA, high altitude; LLS, Lake Louise Score.

**Table 2:** Baseline demographic characteristics in AMS (+) and AMS (−) subjects after arriving at 4400 m.

	Old LLS		*p* value	New LLS		*p* value
	AMS (+) (*n* = 277)	AMS (−) (*n* = 440)		AMS (+) (*n* = 257)	AMS (−) (*n* = 460)	
Age, years	21.44 ± 3.02	21.27 ± 2.90	0.446	21.46 ± 3.07	21.271 ± 2.87	0.398
BMI, kg.m^−2^	21.22 ± 1.89	21.19 ± 1.84	0.849	21.22 ± 1.90	21.19 ± 1.84	0.804
Nationality, *n* (%)			0.131			0.156
Han people	249 (89.9%)	394 (89.5%)		230 (89.5%)	413 (89.8%)	
Tibetan	2 (0.7%)	12 (2.7%)		2 (0.8%)	12 (2.6%)	
Others	23 (9.4%)	34 (7.7%)		25 (9.7%)	35 (7.6%)	
Smoking, *n* (%)			0.294			0.214
Non	71 (25.0%)	123 (28.0%)		66 (25.7%)	128 (27.8%)	
Previous	60 (21.7%)	111 (25.5%)		54 (21.0%)	117 (25.4%)	
Current	146 (52.7%)	206 (46.8%)		137 (53.3%)	215 (46.7%)	
History of HA exposure within 1 year, *n* (%)			0.980			0.866
Yes	43 (15.5%)	68 (15.5%)		39 (15.2%)	72 (15.7%)	
No	234 (84.5%)	372 (84.5%)		218 (84.8%)	388 (84.3%)	
Ascent rate, *n* (%)			0.015			0.004
Fast	140 (50.5%)	263 (59.8%)		126 (49.0%)	277 (60.2%)	
Slow	137 (49.5%)	177 (40.2%)		131 (51.0%)	183 (39.8%)	
Education, *n* (%)			0.744			0.613
University	56 (20.2%)	79 (18.0%)		53 (20.6%)	82 (17.8%)	
High school	153 (55.2%)	252 (57.3%)		140 (54.5%)	265 (57.6%)	
Under high school	68 (24.5%)	109 (23.7%)		64 (24.9%)	113 (24.6%)	

Values are mean ± SD or *n* (%); AMS, acute mountain sickness; BMI, Body Mass Index; HA, high altitude; LLS, Lake Louise Score.

### Risk factors for AMS

The results from logistic regression suggested that ascent protocol was the most important factor affecting the prevalences of AMS. Irrespective of whether the old and new LLS was used for AMS diagnosis, after adjusting the confounders, multivariate regression demonstrated that the fast ascent was a risk factor (OR: 1.52, *p* = 0.006; OR: 1.47, *p* = 0.015) at 3650 m but became a protective factor (OR: 0.67, *p* = 0.018; OR: 0.63, *p* = 0.008) at 4400 m (Tables 3, 4).

**Table 3:** Risk factors of old LLS-AMS and new LLS-AMS after arriving at 3650 m.

	Old LLS		New LLS	
	Univariate	Multivariate	Univariate	Multivariate
Age	0.99 (0.94, 1.04)	0.99 (0.93, 1.05)	0.96 (0.91, 1.01)	0.96 (0.90, 1.02)
BMI	1.02 (0.94, 1.11)	1.03 (0.94, 1.12)	0.98 (0.90, 1.07)	1.01 (0.92, 1.10)
Smoking				
Non	1	1	1	1
Previous	1.03 (0.67, 1.58)	0.99 (0.64, 1.53)	1.27 (0.82, 1.98)	1.19 (0.76, 1.87)
Current	1.05 (0.73, 1.51)	1.01 (0.70, 1.46)	1.18 (0.80, 1.72)	1.12 (0.76, 1.66)
Nationality				
Han people	1	1	1	1
Tibetan	0.35 (0.08, 1.56)	0.35 (0.08, 1.58)	0.42 (0.09, 1.87)	0.41 (0.09, 1.86)
Others	1.26 (0.73, 2.16)	1.36 (0.78, 2.37)	1.31 (0.75, 2.28)	1.46 (0.83, 2.57)
Education				
University	1	1	1	1
High school	1.10 (0.74, 1.65)	1.08 (0.69, 1.69)	1.16 (0.76, 1.78)	1.00 (0.63, 1.59)
Under high school	1.11 (0.70, 1.78)	1.01 (0.61, 1.69)	1.24 (0.77, 2.02)	0.97 (0.57, 1.65)
HA exposure History	1.09 (0.72, 1.64)	1.08 (0.70, 1.65)	1.12 (0.73, 1.71)	1.14 (0.74, 1.77)
Ascent rate (fast vs. slow)	1.50 (1.10, 2.04)*	1.52 (1.11, 2.08)**	1.47 (1.07, 2.02)*	1.47 (1.06, 2.03)*

Values are β/OR (95%CI); *p-value < 0.05. **p-value < 0.01. AMS, acute mountain sickness; BMI, Body Mass Index; HA, high altitude; LLS, Lake Louise Score.

**Table 4:** Risk factors of old LLS-AMS and new LLS-AMS after arriving at 4400 m.

	Old LLS		New LLS	
	Univariate	Multivariate	Univariate	Multivariate
Age	1.02 (0.97, 1.07)	1.01 (0.96, 1.07)	1.02 (0.97, 1.08)	1.01 (0.96, 1.07)
BMI	1.01 (0.93, 1.09)	0.99 (0.91, 1.08)	0.98 (0.92, 1.05)	0.99 (0.91, 1.08)
Smoking				
Non	1	1	1	1
Previous	0.94 (0.61, 1.44)	1.01 (0.65, 1.57)	0.90 (0.58, 1.39)	0.97 (0.62, 1.53)
Current	1.23 (0.86, 1.76)	1.29 (0.90, 1.87)	1.24 (0.86, 1.78)	1.31 (0.90, 1.91)
Nationality				
Han people	1	1	1	1
Tibetan	0.26 (0.06, 1.19)	0.24 (0.05, 1.10)	0.30 (0.07, 1.35)	0.28 (0.06, 1.25)
Others	1.21 (0.71, 2.07)	1.11 (0.64, 1.92)	1.28 (0.75, 2.20)	1.15 (0.66, 2.00)
Education				
University	1	1	1	1
High school	0.86 (0.58, 1.27)	0.85 (0.55, 1.31)	0.82 (0.55, 1.25)	0.82 (0.53, 1.27)
Under high school	1.88 (0.56, 1.36)	0.87 (0.53, 1.43)	0.88 (0.55, 1.39)	0.87 (0.53, 1.45)
HA exposure history	1.01 (0.66, 1.52)	1.13 (0.73, 1.74)	0.96 (0.63, 1.47)	1.10 (0.70, 1.70)
Ascent rate (fast vs. slow)	0.69 (0.51, 0.93)*	0.67 (0.49, 0.92)*	0.64 (0.47, 0.86)**	0.63 (0.45, 0.86)**

Values are β/OR (95%CI); *p-value < 0.05. **p-value < 0.01. AMS, acute mountain sickness; BMI, Body Mass Index; HA, high altitude; LLS, Lake Louise Score.

### Dynamics of AMS

Propensity score-matching was employed to further determine the relationship between ascent protocol and AMS, rigorous adjustment for significant variation among the baseline demographic characteristics was performed to reduce the effect of potential confounding in our study. A 1:1 nearest-neighbor matching without replacement was performed with a caliper width of 0.10. Five hundred and forty-four subjects with a 1:1 ratio of the fast and slow ascent cohorts were included finally. And there were no significant differences in the baseline demographic characteristics between the two cohorts (Supplementary Table 1).

**Figure 2:**
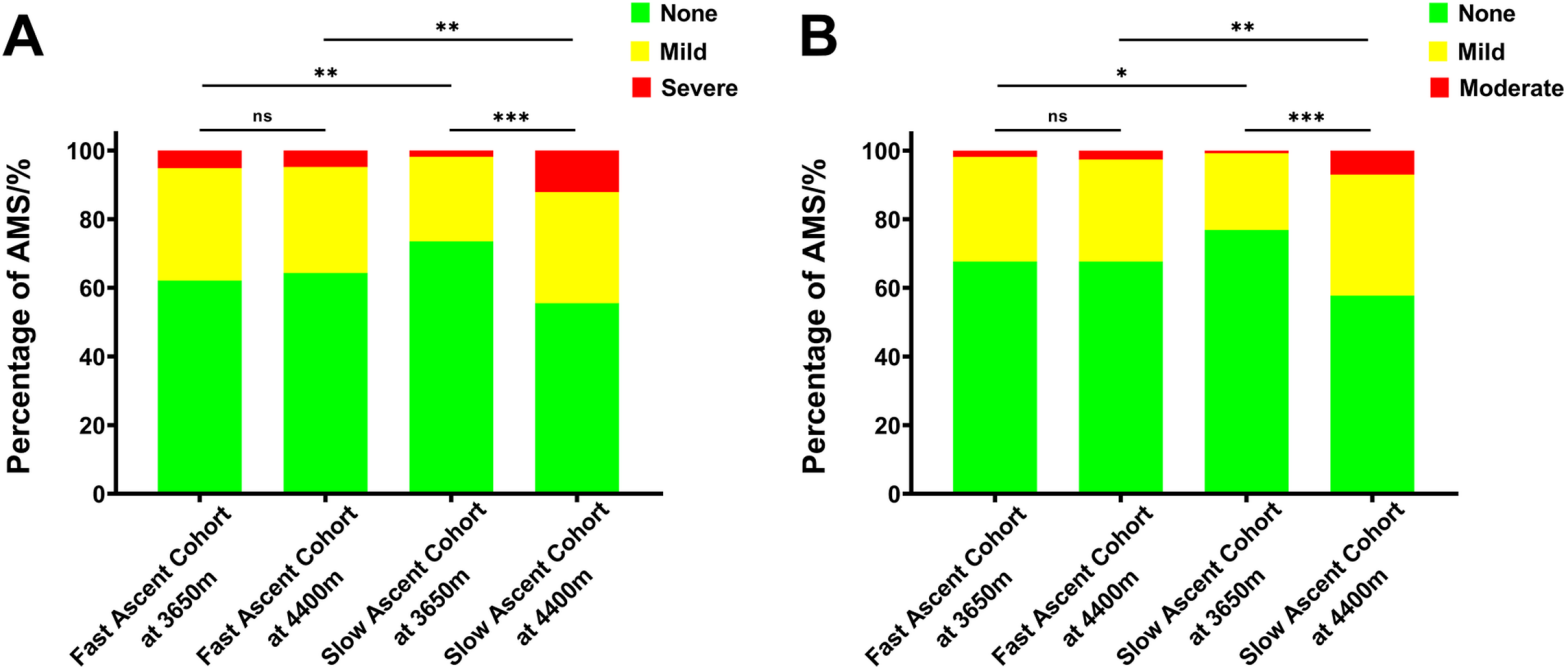
The incidence of AMS according to the different high altitudes in the propensity score-matched cohorts. **(A)** AMS diagnosed by the old LLS and **(B)** AMS diagnosed by the new LLS. AMS, acute mountain sickness; LLS, Lake Louise Score.

In the fast ascent cohort, 103 subjects (37.9%) and 88 subjects (32.4%) experienced AMS following the initial ascent to 3,650 m, using the old and new LLS for diagnosis, respectively. And the number of AMS patients became 97 (35.7%) and 88 (32.4%) after further ascent to 4,400 m when diagnosed by the old and new LLS, respectively. In contrast, in the slow ascent cohort, 72 subjects (26.5%) and 63 subjects (23.2%) developed AMS after the initial ascent to 3,650 m using the old and new LLS for diagnosis, respectively. However, upon further ascent to 4,400 m, the number of AMS patients increased to 121 (44.5%) and 115 (42.3%) when diagnosed by the old and new LLS, respectively (Table 5; Figure 2).

**Table 5:** Incidence of clinical indicators and outcomes at different altitudes in the fast or slow ascent cohort.

	Fast ascent cohort		Slow ascent cohort		P1	P2	P3	P4
	3,650 m (*n* = 272)	4,400 m (*n* = 272)	3,650 m (*n* = 272)	4,400 m (*n* = 272)				
Old LLS-AMS rate	103 (37.9%)	97 (35.7%)	72 (26.5%)	121 (44.5%)	0.594	<0.001	0.004	0.036
Old LLS-AMS score	2.41 ± 1.90	2.15 ± 1.94	1.71 ± 1.65	2.60 ± 2.35	0.113	<0.001	<0.001	0.015
New LLS-AMS rate	88 (32.4%)	88 (32.4%)	63 (23.2%)	115 (42.3%)	1.000	<0.001	0.017	0.017
New LLS-AMS score	2.00 ± 1.54	1.82 ± 1.62	1.50 ± 1.41	2.22 ± 1.90	0.185	<0.001	<0.001	0.008
Clinical indicators								
SBP, mmHg	120.61 ± 11.59	119.11 ± 11.02	116.67 ± 10.31	124.57 ± 11.68	0.122	<0.001	<0.001	<0.001
DBP, mmHg	79.58 ± 9.83	77.82 ± 9.54	75.28 ± 9.80	81.61 ± 19.80	0.035	<0.001	<0.001	<0.001
Pulse rate, beat/min	82.64 ± 11.99	82.11 ± 12.58	79.18 ± 11.15	85.53 ± 11.17	0.613	<0.001	<0.001	<0.001
SpO_2_, %	89.34 ± 3.19	87.32 ± 2.90	91.69 ± 2.19	84.97 ± 2.60	<0.001	<0.001	<0.001	<0.001
Clinical symptoms								
Headache	123 (45.2%)	109 (40.1%)	120 (44.1%)	151 (55.5%)	0.225	0.008	0.796	<0.001
Dizziness	140 (51.5%)	128 (47.1%)	135 (49.6%)	158 (58.1%)	0.303	0.048	0.668	0.010
GI upset	71 (26.1%)	64 (23.5%)	37 (13.6%)	68 (25.0%)	0.487	<0.001	<0.001	0.689
Insomnia	95 (34.9%)	82 (30.1%)	52 (19.1%)	77 (28.3%)	0.234	0.012	<0.001	0.637
Fatigue	169 (62.1%)	148 (54.4%)	101 (37.1%)	150 (50.3%)	0.068	<0.001	<0.001	0.863
Paraesthesia	85 (31.3%)	72 (26.5%)	74 (27.2%)	92 (33.8%)	0.219	0.094	0.300	0.062
Constipation	44 (16.2%)	31 (11.4%)	34 (12.5%)	48 (17.6%)	0.106	0.093	0.271	0.039
Dyspnea	95 (34.9%)	80 (29.4%)	23 (8.5%)	92 (33.5%)	0.169	<0.001	<0.001	0.269
Cough	101 (37.1%)	94 (34.6%)	21 (7.7%)	97 (35.7%)	0.531	<0.001	<0.001	0.788
Chest distress	94 (34.6%)	83 (30.5%)	49 (18.0%)	89 (32.7%)	0.314	<0.001	<0.001	0.580
Palpitation	52 (19.1%)	43 (15.8%)	30 (8.7%)	54 (19.9%)	0.309	0.004	<0.001	0.218
Tinnitus	96 (35.3%)	81 (29.8%)	56 (20.6%)	84 (30.9%)	0.265	0.006	<0.001	0.780
Dazzling	53 (19.5%)	47 (17.3%)	20 (7.4%)	63 (23.2%)	0.507	<0.001	<0.001	0.088
Lethargy	79 (29.0%)	61 (22.4%)	39 (14.3%)	63 (23.2%)	0.118	0.015	<0.001	0.838
Activity reduction	155 (57.0%)	140 (51.5%)	65 (23.9%)	151 (55.5%)	0.730	<0.001	<0.001	0.344

Values are *n* (%). P1 value for comparison between 3,650 m and 4,400 m in the fast ascent cohort. P2 value for comparison between 3,650 m and 34,400 m in the slow ascent cohort. P3 value for comparison between fast and slow ascent cohort at 3650 m. P4 value for comparison between fast and slow ascent cohort at 4400 m. GI upset = Gastrointestinal upset.

Furthermore, after reaching an intermediate altitude (3,650 m), it was evident that the incidence of AMS (37.9% vs. 26.5%, *p* = 0.004; 32.4% vs. 23.2%, *p* = 0.017) and its corresponding scores (2.41 ± 1.90 vs. 1.71 ± 1.65, *p* < 0.001; 2.00 ± 1.54 vs. 1.50 ± 1.41, *p* < 0.001) were notably higher in the fast ascent cohort compared to the slow ascent cohort, using the old and new LLS for diagnosis, respectively. However, upon further ascent to the higher altitude (4,400 m), the slow ascent cohort exhibited significantly higher incidence (35.7% vs. 44.5%, *p* = 0.036; 32.4% vs. 42.3%, *p* = 0.017) and severity (2.15 ± 1.94 vs. 2.60 ± 2.35, *p* = 0.015; 1.82 ± 1.62 vs. 2.22 ± 1.90, *p* = 0.008) of AMS compared to the fast ascent cohort when diagnosed by the old and new LLS, respectively. These results indicated that prevalence of AMS, as well as its evolution were shaped and influenced by ascent protocol (Table 5; Figure 2).

### Dynamics of clinical symptoms

Among the 15 clinical symptoms that developed, fatigue (62.1%) was most common in the fast ascent cohort after the initial ascent to 3,650 m, followed by activity reduction, dizziness, headache, and cough. And the order of the first five clinical symptoms was not markedly changed after further ascent to 4,400 m. Interestingly, dizziness (49.6%) was most common in the slow ascent cohort after the initial ascent to 3,650 m, followed by headache, fatigue, paraesthesia, and activity reduction. However, after the further ascent to 4,400 m, dizziness (42.4%) was the leading symptom, followed by activity reduction, headache, fatigue and cough (Table 5).

The results from the symptomatic analysis showed that the incidences of clinical symptoms including headache, dizziness, GI upset, insomnia, fatigue, dyspnoea, cough, chest distress, palpitation, dazzling, lethargy and activity reduction in subjects who ascended to the higher altitude from an intermediate altitude were significantly increased in the slow ascent cohort. Nevertheless, in the fast ascent cohort, almost all clinical symptoms were even decreased after further ascent to a higher altitude, except for constipation and paraesthesia. But the difference did not reach statistical significance. Additionally, the incidences of AMS-related symptoms, including GI upset (26.1% vs. 13.6%, *p* < 0.001), insomnia (34.9% vs. 19.1%, *p* < 0.001) and fatigue (62.1% vs. 37.1%, *p* < 0.001) in the subjects quickly ascended to an intermediate altitude, were higher than those who slowly ascended to an intermediate altitude. However, after ascending to the higher altitude, the incidences of headache (40.1% vs. 55.5%, *p* < 0.001) and dizziness (51.5% vs. 58.1%, *p* = 0.010) were significantly higher in the slow ascent cohort than in the fast ascent cohort. These results elucidated that the clinical features of AMS, as well as their dynamics were shaped and influenced by ascent protocol (Table 5).

## Discussion

Our findings showed that: (1) In the fast ascent cohort, the incidence of AMS was 37.9 and 32.4% at 3650 m, which decreased to 35.7 and 32.4% when further ascending to 4,400 m, as diagnosed by the old and the new LLS, respectively; (2) In the slow ascent cohort, the incidence of AMS was 26.5 and 23.2% at 3650 m, which increased to 44.5 and 42.3% when further ascending to 4,400 m, as diagnosed by the old and the new LLS, respectively; (3) the ascent protocol was the primary factor that affected the prevalence of AMS and related symptoms, showing that the slow ascent protocol was protective during the initial ascent but became a risk factor after further ascending to a higher altitude.

AMS is a prevalent medical condition that significantly impacts the well-being of a substantial portion of the population (2). A comprehensive systematic review synthesized the reported occurrences of AMS across 53,603 individuals, revealing a median incidence rate of 60% in randomized trials, 51% in cohort studies, and 32% in cross-sectional studies. Furthermore, the findings from the multivariate analysis showed that the significant correlation between AMS and some factors, including study design, mode of ascent, peak altitude attained and demographics (17). Previous results showed that the overall incidence of AMS was 53% after ascending to 4,243 m altitude in the Himalayas of Nepal (19). It had also been reported that the prevalence of acute mountain sickness was 9% at 2850 m, 13% at 3050 m, and 34% at 3650 m (20); the latter of the three values was comparable with our prevalence in the slow ascent cohort at 3650 m. Indeed, the prevalence of AMS is primarily controversial because it could be influenced by various confounding factors. The incidence of AMS among tourists who ascend directly to 3,740 meters by airplane is significantly higher than among those who ascend to the same altitude by foot (17), which was consistent with our present results, indicating that individuals who ascend to HA by airplane experience AMS more frequently than those who follow alternative protocols of ascent.

Upon reaching a new altitude exceeding 2,500 m, individuals who have not undergone acclimatization typically exhibit a constellation of non-specific symptoms. Among these diverse manifestations, headache stands as the pivotal and crucial symptom for diagnosing AMS (21, 22). After sequencing the 15 clinical symptoms reported in our present study, the main symptoms of AMS, including headache and dizziness and fatigue, remained as the cardinal symptoms after arriving HA, which was consistent with previous results (23). It had also been demonstrated that the most common AMS symptom was headache, followed by fatigue of children trekking at 3952 m (24). However, in the slow ascent cohort, AMS-related symptoms were seemingly not the leading symptoms, and their incidence was also lower than that in the fast ascent cohort after ascending to HA, which suggested that the ascent protocol might hold an important effect on the clinical symptoms in subjects further ascended to a higher altitude after a short term of staying at the intermediate altitude. Additionally, activity reduction, which was not a symptom for AMS diagnosis, was another more frequent complaint. Consistently, a previous review claimed that individuals with AMS were frequently incapacitated (4). Although insomnia had been excluded from the newly revised AMS diagnostic criteria (6), the incidence of insomnia was still prevalent, as previously described (25), and was more frequent in the fast ascent cohort than in the slow ascent cohort in our study.

The incidence of AMS exhibited a strong correlation with the rate of ascent and the individual’s age, whereas no significant correlation was observed with gender or previous altitude exposure (19). However, the influence of the latter two on AMS is still controversial (26, 27). In addition, differences in individual susceptibility caused by ethnic and genetic factors also affect the AMS incidence (14, 28). A previous systematic review summarized that the fast ascent protocol had an OR of 4.69, whereas a slow ascent protocol had an OR of 0.30 for the incidence of AMS (5), which was highly consistent with our present results during the initial ascent to 3,650 m by plane or by bus, respectively. Moreover, a recent review conducted by Burtscher et al. examined the AMS incidence in 11,021 individuals who ascended to various altitudes ranging from 2,200 to 4,559 m, revealed an impressive 4.5-fold steeper increase in the AMS incidence for air travel as compared with slower ascent modes (hiking or combined other modes of transportation). Nonetheless, the incidence of AMS among subjects ascending by airplane was notably higher, which may be attributed to the predominantly male cohort in our study. Furthermore, a recent meta-analysis has failed to yield consistent findings indicating whether cigarette smoking served as a preventive measure or a contributing factor to AMS (29). Our data suggested that compared with non-smokers, smokers did not exhibit a significant difference of AMS among different ascent protocols, altitudes attained and diagnostic criteria.

It is advisable to prevent AMS by ascending gradually at altitudes exceeding 3,000 m and incorporating a rest day every 3–4 days (4, 30). This protocol, known as “staged ascent,” has been widely regarded as the optimal strategy for preventing AMS (30). A previous study revealed that staging at an intermediate altitude for 2 days led to a decrease in the occurrence of AMS when compared to individuals who ascended directly (31). Nevertheless, these strategies failed to mitigate the risk of AMS when a swift ascent to higher altitudes was undertaken (32, 33). Thus, the results from studies aimed at determining the advantages of intermittent hypoxic exposures for preventing AMS were somewhat conflicting for subjects intending to ascend to even higher altitudes. Our results from the field study conducted at HA suggested that the slow ascent protocol accompanied by a two-day staging period at an intermediate altitude did not mitigate the occurrence of AMS subsequent to ascending to above 4,000 m. Results from a recent study also indicated that 2 days of staging at 2500 and 3,500 m was not protected against AMS but 3,000 m was the optimal 2-day staging altitude to induce acclimatization and provide AMS protection following subsequent ascent to 4,300 m (18). Further exploration is required to develop more effective AMS pro-acclimatization strategies for subsequent ascents. Interestingly, despite a notably high incidence of AMS among subjects who underwent a rapid ascent to the initial altitude by plane, the number of subjects experiencing AMS after ascending to an even higher altitude was significantly lower than those who underwent the initial ascent in the fast ascent cohort and those in the slow ascent cohort during their subsequent ascent. Initial rapid ascent, coupled with intermediate altitude acclimatization, may facilitate physiological adaptation for subsequent ascents which was associated with arterial oxygen saturation (18). These findings might imply a novel strategy for AMS prevention in individuals traveling to or working at HA when a further ascent to a higher altitude was needed.

## Limitations

There were still several limitations. First, the enrolled participants were young, healthy men, and whether the established results can extend to other types of individuals (such as women and mountain climbers) are still unknown. Second, due to a lack of exercise, which is a known trigger for AMS, the severity of AMS in this cohort is mild. Whether these findings can be replicated under conditions of higher intensity physical activity (such as sports) at high altitudes remains to be confirmed. Third, the categorization of AMS relied solely on self-reporting without immediate medical verification, potentially lead to classification bias. Finally, although the incidence and severity of AMS in the fast ascent group were greater than those in the slow ascent group at 3650 m, subjects from the fast ascent group underwent a longer period of acclimatization. Considering the time-dependent nature of acclimatization, this may have an impact on disease status during further ascent.

## Conclusion

To the best of our knowledge, this is the first study to investigate the dynamics in the prevalence and clinical manifestations of AMS during ascent to HA. Moreover, we found that the ascent protocol was the primary factor that affected the prevalence of AMS and related symptoms, showing that a fast ascent protocol, which acted as a risk factor during the initial ascent, was converted into a protective factor after further ascending to a higher altitude. Initial fast ascent combined with intermediate altitude acclimatization may facilitate physiological adaptation (improving oxygenation condition) for subsequent ascent to higher altitudes. However, the fundamental mechanisms behind this process require further elucidation. These findings might imply a novel strategy for HA travels or work with a plan to further ascend to a higher altitude.

## Data Availability

The original contributions presented in the study are included in the article/Supplementary material, further inquiries can be directed to the corresponding authors.
