# Supplementary material for: Dynamics in the prevalence and clinical manifestations of acute mountain sickness of different ascent protocols during high altitudes exposure
Source: Front Public Health. 2024 Nov 21;12:1472935. doi: 10.3389/fpubh.2024.1472935 (PMC11617577; doi:10.3389/fpubh.2024.1472935)
Supplement: Supplementary file 1 [file Table_1.DOC]

| **Supplemental Table 1. Baseline Demographic Characteristics of the Propensity Score-Matched Subjects in the Fast or Slow Ascent Cohort** | | | |
| --- | --- | --- | --- |
|  | Fast Ascent Cohort (n=272) | Slow Ascent Cohort (n=272) | P value |
| **Age, years** | 21.17±2.45 | 21.06±2.71 | 0.608 |
| **BMI, kg.m^-2^** | 21.19±1.86 | 21.19±1.68 | 0.974 |
| **Nationality, n (%)** |  |  | 0.829 |
| Han people | 244 (89.7%) | 247 (90.8%) |  |
| Tibetan | 7 (2.6%) | 5 (1.8%) |  |
| Others | 21 (7.7) | 20 (7.4%) |  |
| **Smoking, n (%)** |  |  | 0.870 |
| non | 73 (26.8%) | 76 (27.9%) |  |
| previous | 64 (23.5%) | 59 (21.7%) |  |
| current | 135 (49.6%) | 137 (50.4%) |  |
| **History of HA exposure within 1 year, n (%)** | | | 0.767 |
| yes | 26 (9.6%) | 24 (8.8%) |  |
| no | 246 (90.4%) | 248 (91.2%) |  |
| **Education, n (%)** |  |  | 0.572 |
| university | 48 (17.6%) | 42 (15.4%) |  |
| high school | 154 (56.6%) | 166 (61.0%) |  |
| under high school | 70 (25.7%) | 64 (23.5%) | |  |  | | --- | --- | |
| Values are Mean ± SD or n (%); Abbreviations as in Table 1; Subjects in the 2 cohorts were matched for all demographic characteristics by using propensity score. | | | |
